# Co-Designing a Justice-Oriented Assessment System in a Pediatric Residency Program: Report from the Designing for Equity in Medical Education Project

**DOI:** 10.5334/pme.1541

**Published:** 2025-04-07

**Authors:** Hannah Kakara Anderson, Pricilla Cabral, Emma Gerstenzang, Christine Liverpool, Marciel Gonzalez, Anna Weiss, Danielle Cullen, Dorene Balmer, Marjan Govaerts, Daniel C. West, Jamiu Busari

**Affiliations:** 1 University of Pennsylvania Perelman School of Medicine, US; 2 The Children’s Hospital of Philadelphia, US; 3 Maastricht University School of Health Professions Education, NL

## Abstract

**Background and Need for Innovation:**

There is a large body of evidence that assessment systems in medical education are inequitable for many groups of learners. A common approach to improve equity has been the use of organizational strategies, where training program leaders work to develop and implement improvements in existing assessment systems from their perspective to improve equity. However, emerging assessment approaches, such as justice-oriented assessment, argue that assessment systems must be made more equitable by critique and re-building through co-design with learners, assessors, and other key users. Little is known about how to apply these methods to workplace-based assessment in medical education.

**Goal of Innovation:**

To fill the knowledge gap about how to co-design a more equitable, justice-oriented, workplace-based assessment system in pediatric post-graduate medical education.

**Steps taken for Development and Implementation of innovation:**

Using the Design Justice framework, the authors completed 4 of the 5 phases of Design Thinking to co-design with learners and other users a workplace-based assessment system in their institution’s pediatric residency program.

**Evaluation of Innovation:**

To understand whether and how Design Justice principles were present and operationalized in the process of co-designing the assessment system, the authors evaluated the design activities in each phase of the Design Thinking process, the outputs of the design process, and the experiences of participating users.

**Critical Reflection:**

Evidence of Design Justice principles included participants’ feelings of being heard, affirmed, and empowered, as well as the design teams’ iterative, critical reflection on making the project accessible, accountable, sustainable, and collaborative. This project offers a practical example of co-designing a justice-oriented assessment system, the process and principles of which can inform the efforts of advancing equity in assessment.

## Background and Need for Innovation

There is a large body of evidence that assessment systems in medical education are inequitable for learners who are minoritized by their race, gender, and other characteristics [1,2,3,4]. These inequities persist largely because there are critical gaps in our knowledge about how to design more equitable assessment systems [5,6]. A common approach to fill these gaps has been the use of organizational strategies, in which training program leaders apply a standardized checklist, guide, or other model to evaluate their assessment systems against a comprehensive set of pre-determined best-practices [7]. Based on this evaluation, program leaders adjust assessment systems accordingly. Although the application of these strategies has resulted in some improvements in the overall quality of assessment in medical education, the effectiveness of this approach has been limited because it focuses on the application of decontextualized and standardized best practice models [6]. Thus, these strategies may overlook important aspects of local context that can impact equity [8].

Justice-oriented assessment is an alternative approach in which the path to achieving equity in assessment is to strive for justice. For clarity, we define equity as the treatment, access, and opportunities that are appropriate, responsive, individualized, and sustaining. We define justice as repairing, restoring, or transforming specific underlying, existing harms and disparities that cause inequity. A key distinction is that organizational strategies (as described above) are decontextualized and intended to be used and led by program leaders, while justice-oriented assessment takes an explicitly antiracist and anti-oppressive stance by critiquing, reimagining, and redesigning assessments from the perspective of marginalized users, particularly learners [9,10].

In justice-oriented assessment, assessment systems are critiqued and re-built through *co-design* by diverse users (e.g., learners, assessors, etc.) in the context of their local programs, rather than evaluated and adjusted by program leaders using pre-existing standards or models [11]. Attention to local context is critical for developing a fuller understanding of local issues impacting equity and ensuring successful implementation of re-designed assessment systems [5]. This “contextual fit” is crucial because training programs exist in diverse contexts with diverse users; therefore, justice-oriented assessment argues that assessment systems must respond to specific local needs and harms.

One approach to completing a co-design process is by using the process of Design Thinking (DT). DT typically involves five flexible steps of collaboration between key users to generate solutions that meet their needs and fit context, i.e. 1) empathize with users; 2) define the problem; 3) ideate to generate ideas; 4) prototype to co-create solutions; and 5) test solutions) [12]. DT is increasingly recognized as a practical approach for addressing a variety of community and educational problems [13,14,15].

However, using DT alone is not sufficient to ensure that a co-design process will result in more equitable solutions [16,17]. Thus, justice-oriented designers have developed Design Justice, a set of accountability principles that act as theoretical and practical guardrails for the design process. These principles guide decision-making and activities in the design process to ensure that the design process, and any solutions generated by it, are just. The Design Justice Network Principles (Chart 1) can be applied to the DT process and to orient the thinking, behaviors, and choices of those who participate in the design process to practice justice at every stage of a design project [18].

**Chart 1:** Design Justice Principles.


1. We use design to sustain, heal, empower our communities, and seek liberation from exploitative and oppressive systems.

2. We center the voices of those directly impacted by the outcomes of the design process.

3. We prioritize design’s impact on the community over the designer’s intentions.

4. We view change as emergent from an accountable, accessible, and collaborative process rather than as a point at the end of a process.

5. We see the role of the designer as a facilitator rather than an expert.

6. We believe that everyone is an expert based on their own lived experience, and we all have unique and brilliant contributions to bring to a design process.

7. We share design knowledge and tools with our communities.

8. We work towards sustainable, community-led and -controlled outcomes.

9. We work towards non-exploitative solutions that reconnect us to the earth and each other.

10. Before seeking new design solutions, we look for what already works at the community level. We honor and uplift traditional, indigenous, and local knowledge and practices.


Justice-oriented assessment has been successfully applied to co-design more equitable assessment systems in primary, secondary, and higher education in the United States [19]. However, we currently know little about how to apply these types of justice-oriented assessment methods to co-design assessment systems in medical education. This knowledge gap is important to fill because using justice-oriented assessment in medical education can advance the equity of assessment systems in a way that we currently cannot, using organizational strategies alone.

## Goal of Innovation

To fill the knowledge gap about how to co-design a testable prototype of a justice-oriented assessment system in graduate medical education using an innovative application of Design Thinking guided by Design Justice.

## Steps taken for Development and Implementation

### Context

This project took place in the 3-year, general pediatrics residency program of a large, urban, academic, free-standing children’s hospital in the United States that serves a large local patient community ([55.8%] identify as Black/African American or Hispanic/Latine) and a national/international community of patients. The residency program has approximately 170 residents (20% self -identified as underrepresented in medicine [Black/African American, Hispanic/Latine, and American Indian, Native Hawaiian or Pacific Islander].

Within the residency program’s assessment system, 44 assessment forms (housed in a web-based electronic platform) were used by assessors to evaluate residents in 44 clinical rotations or other training experiences (e.g., inpatient general pediatrics, ambulatory experiences, and specialty electives). Assessors included attending physicians (fully trained physicians who are responsible for both ensuring appropriate patient care and supervising residents caring for patients), senior residents (near-peer resident physicians who are 1–2 years further advanced in residency training and provide direct supervision of residents with less training), and peer residents (residents in the same year of training assigned to the same training experience). Administrative staff assigned assessments based on training schedules so they could be sent, recorded, stored, and accessed within the web-based system. Based on these assignments, the system sent automated emails to assessors midway and after each training experience to prompt them to complete assessment forms. In training settings where residents and assessors changed very frequently (e.g., emergency department), assessors received assessment forms at the end of each shift. Residents could read/access these assessments only when three or more were submitted. Before Clinical Competency Committee (CCC) meetings, resident mentors and the CCC members, comprising all the associate program directors and the residency program director, reviewed assessment summary reports without resident involvement. The CCC met quarterly.

This project was reviewed and deemed exempt by the Institutional Review Board at the Children’s Hospital of Philadelphia.

### The Design Process

Using steps 1–4 of the Design Thinking process, we worked with users to co-design a testable prototype of a new assessment system. We applied Design Justice principles to guide decision-making and all activities. Step 5 of Design Thinking – implementing and testing the prototype – is outside the scope of this paper and will be reported in future papers.

**Step 1 (Empathize):** We conducted semi-structured interviews with all user groups to understand their perspectives on equity in assessment. We also conducted brief unstructured observations of program staff, assessors, and residents to understand the workflow of assessments within the web-based system (e.g., sending notifications to complete assessments, accessing assessments). We created our interview guides using concepts from the Anti-deficit Model for Equitable Assessment. [Supplement 1: Interview Guide] Interviews and observations were audio-recorded, transcribed, and de-identified. We collected and de-identified documents, including all assessment forms and local training program policy around assessment. We then created empathy maps to serve as a visual tool for understanding the needs of each user group. [Supplement 2: Users and Outputs].

**Step 2 (Define):** We held three design sessions with users to define local equity issues in assessment: (1) orienting them to the DT process, (2) discussing empathy maps, (3) reflecting on shared experiences and values, and (4) building consensus on harmful and beneficial aspects of assessment.

**Step 3 (Ideate):** We conducted two design sessions to co-create design principles for an equitable assessment system. Outputs from Steps 1 and 2 informed discussions. Users worked in homogeneous and mixed groups to envision an equitable assessment system in line with design justice. By way of example, the design principle that stresses interdependence and mutuality reflects on Design Justice principles that prioritize the community and defines change as a collaborative process. Informed by session artifacts and transcripts, HA and DB constructed design principles, which were then reviewed by user representatives EG and CL.

**Step 4 (Prototype):** We held two design sessions to create a prototype assessment system using the principles from Step 3. Users engaged in a card-sorting activity to organize and generate new assessment components. They critiqued, redesigned, and assembled prototype components in alignment with the design principles from Step 3.

All sessions in Steps 2–4 were facilitated by HA, PC, and DB. Sessions were audio-recorded. Participants were invited to complete an optional Demographic Survey and submit anonymous feedback cards at the end of each session.

## Evaluation of Innovation

### Goal of evaluation

We sought to understand whether and how Design Justice principles were present and operationalized in our design process practices, the design process outputs, and the design participants’ experiences. Reporting project outcomes (i.e., the structure and impacts of implementing the prototype assessment system itself) is outside the scope of this manuscript; however, in design-based research, reporting on the design process practices and outputs, the experiences of design participants, and the extent to which we practiced the design principles (theoretical and practical underpinnings) during the design process are as important as the project outcomes [20]. Although the outcomes of a locally specific prototype system may not be transferable to another context/institution, knowledge about design processes can be applied and replicated across contexts and inform the work of others faced with similar problems but different contexts [21].

### Evaluation Data and Analysis

We recorded the number of design sessions held and the number and demographics of participants for each step of the DT process. To ensure we included a diverse range of users in the design process, we collected information about self-identification of race, ethnicity, and gender identity for all design participants. We summarized these data using descriptive statistics in Microsoft Excel.

In addition to interview, observation, and document data (e.g., assessment forms) collected during Step 1, we systematically collected data from all design sessions in Steps 2–4 via facilitator notes, de-identified transcripts of design sessions, any outputs/artifacts (pictures, collages, sticky notes, etc.) produced during the sessions, and anonymous feedback cards with answers to session evaluation questions. Two authors (HA, PC) organized all data into Atlas.ti (Scientific Software, Berlin) at the time of collection and then used deductive content analysis using Design Justice principles as codes to code the data. At weekly meetings, authors HA, PC, DW, and DB discussed processes and data analyses, making iterative improvement adjustments. At quarterly meetings, authors EG, CL (internal user representatives) met with HA to review and reflect on design activities and outputs. At the end of step 4, HA and DB met with external user authors MG and JB to reflect and prepare summary figures/tables.

## Critical Reflection

**Figure 1:**
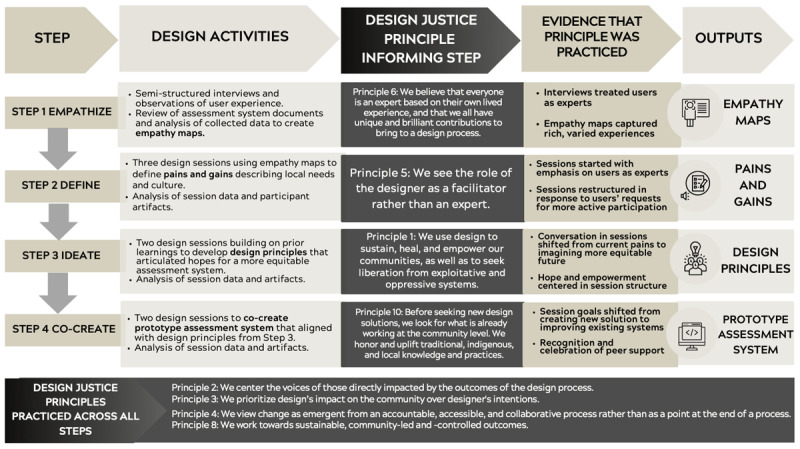
Steps of the Co-Design Process: Design Activities in each Step, Design Justice Principle informing each Step, Evidence that Principle(s) was Practiced, and Outputs of Steps. From a Design Process to Co-Design a more Equitable Prototype Assessment System in Pediatrics. 2024.

We found that 8 of 10 Design Justice principles were practiced in the design process with four principles (2, 3, 4, and 8) practiced in all steps. Four principles (1, 5, 6, and 10) were practiced in only one step (Figure 1). Two principles (7 and 9) were not practiced or not evident during the design process. Mindful of this, we are looking for ways to practice these principles more consistently as we complete the planned next step of the Design Thinking process, Step 5 – Implementing and Testing.

### Principles practiced across all steps

#### Principle 2: We center the voices of those directly impacted by the outcomes of the design process

Throughout all steps we purposefully recruited users who identified as minoritized and/or marginalized based on the demographic survey (Supplement 2: Users and Outputs). We also included some users who did not identify as such, because they are directly impacted by any changes to the assessment system. We intentionally centered the voices of marginalized participants by partnering with co-facilitators from user groups (program staff, assessors, or residents) and breaking out into homogeneous groups where users could develop ideas before reporting out in heterogenous large groups to ensure all were acknowledged and reflected in the design. In end-of-session anonymous feedback cards, participants responded to the question “How do you feel leaving this session?” with the words “empowered, “reflective,” and “hopeful.” The most common word used by participants across all design sessions was “heard.” We also encountered pitfalls in our practice of this principle. Despite recruiting a large, diverse group of users, we did not initially include patient and community users in the process until it was brought up by participating residents and leadership. In response, we are working with the Research and Hospital Family Advisory Councils at the institution to engage patients and families to participate in the future.

#### Principle 3: We prioritize design’s impact on the community over the designer’s intentions

In early stages, some leadership users initially expressed disbelief that the training program could be a “community” as referred to in Design Justice. Thus, we intentionally worked to establish a sense of community accountability within each design session in steps 2–4. Each design session opened with the participants and facilitators repeating out loud three community commitments: 1) everybody’s story matters; we will listen to each other’s truths, 2) our actions matter: we will be accountable for our impacts on each other, and 3) we belong to this community: we will care for ourselves and each other.

#### Principle 4: We view change as emergent from an accountable, accessible, and collaborative process rather than as a point at the end of a process

We found evidence of this principle at work in the framing of design sessions *(“We are bringing back our progress so far so you can see what’s happening and hold us accountable”* – facilitator, design session transcript, step 3) and in the continual, iterative nature of our work timelines. We regularly brought updates (as well as actual outputs and deidentified data) back to the training program community in design sessions and via three presentations at committees and work groups and a presentation open to the entire hospital community. We repeated design sessions, sometimes repeating entire steps of the process (as in step 2) at different times, in-person and virtual, and held design sessions during already-set-aside meeting times (e.g., residency noon conference) to make sessions as accessible as possible.

#### Principle 8: We work towards sustainable, community-led and -controlled outcomes

This principle changed how we operated during one critical decision: the decision to eliminate a potential feature of the prototype system – an expiration date for assessment requests – which was led by groups of residents in real time during a step 4 design session, rather than by the project leaders after the design session. In end-of-session anonymous feedback cards, users responded to the question, “What’s one word to describe this session?” with the words, “resident-led,” “thought-provoking,” “collaborative,” “quick,” and “supportive.” The most common word used by users across all sessions was “affirming.”

### Principles not practiced

Despite regularly reviewing all Design Justice principles among our design team, we identified two principles that were not actively practiced during the design process: Principles 7 and 9. This does not necessarily mean that we did not incorporate these principles at all, instead, they did not appear to explicitly inform our actions and decisions when we went back and evaluated the process.

In addition to not explicitly incorporating these two principles, this project has other limitations: first, as with any educational innovation, the project required significant buy-in from leadership. Second, we did not solicit in-depth perspectives from participants – we only collected end-of-session anonymous feedback cards. We plan to conduct in-depth interviews with participants at a later date to more deeply probe for participants’ experiences during the design process.

## Conclusions and Takeaways

We demonstrated that Design Justice can be used to guide the co-design of a justice-oriented assessment system for a pediatric residency program. As medical education programs increasingly try to advance equity in assessment, our work illuminates the importance of not only intending to design a more equitable assessment system but also ensuring an equitable *process* in the act of doing so. Design Justice afforded this project several important things: participants’ feelings of being heard, affirmed, and empowered, as well as the design teams’ iterative, critical reflection on making the project accessible, accountable, sustainable, and collaborative. Although the design output of our project is specific to our context, the design process has the potential to be applied and replicated by others faced with similar problems but different contexts.

## Additional Files

The additional files for this article can be found as follows:

10.5334/pme.1541.s1 Supplement 1. Interview Guide used in a Design Process to Co-Design a more Equitable Prototype Assessment System in Pediatrics. 2024.

10.5334/pme.1541.s2 Supplement 2. Users and Outputs for Each Step of a Design Process to Co-Design a more Equitable Prototype Assessment System in Pediatrics. 2024.

## References

[1] Klein R, Ufere NN, Schaeffer S, et al. Association Between Resident Race and Ethnicity and Clinical Performance Assessment Scores in Graduate Medical Education. Acad Med. 2022; Publish Ahead of Print. DOI: 10.1097/ACM.0000000000004743 PMC9910786 35583954

[2] Klein R, Julian KA, Snyder ED, et al. Gender Bias in Resident Assessment in Graduate Medical Education: Review of the Literature. J Gen Intern Med. 2019;34:712–719. DOI: 10.1007/s11606-019-04884-0 30993611 PMC6502889

[3] Teherani A, Perez S, Muller-Juge V, Lupton K, Hauer KE. A Narrative Study of Equity in Clinical Assessment Through the Antideficit Lens. Acad Med. 2020;95(12S):S121–S130. DOI: 10.1097/ACM.0000000000003690 33229956

[4] Anderson HL, Abdulla L, Balmer DF, Govaerts M, Busari JO. Inequity is woven into the fabric: a discourse analysis of assessment in pediatric residency training. Adv Health Sci Educ. Published online June 23, 2023. DOI: 10.1007/s10459-023-10260-9 37351698

[5] Anderson HL, Kurtz J, West DC. Implementation and Use of Workplace-Based Assessment in Clinical Learning Environments: A Scoping Review. Acad Med. 2021;96(11S):S164–S174. DOI: 10.1097/ACM.0000000000004366 34406132

[6] Kinnear B, Weber DE, Schumacher DJ, Edje L, Warm EJ, Anderson HL. Reconstructing Neurath’s Ship: A Case Study in Reevaluating Equity in a Program of Assessment. Acad Med. 2023;98(8S):S50–S56. DOI: 10.1097/ACM.0000000000005249 37071695

[7] Lucey CR, Hauer KE, Boatright D, Fernandez A. Medical Education’s Wicked Problem: Achieving Equity in Assessment for Medical Learners. Acad Med. 2020;95(12S):S98–S108. DOI: 10.1097/ACM.0000000000003717 32889943

[8] Kakara Anderson HL, Govaerts M, Abdulla L, Balmer DF, Busari JO, West DC. Clarifying and Expanding Equity in Assessment by considering three orientations: fairness, inclusion and justice. Med Educ; 2024. In Production. DOI: 10.1111/medu.15534 PMC11976206 39279355

[9] Randall J. “Color-Neutral” Is Not a Thing: Redefining Construct Definition and Representation through a Justice-Oriented Critical Antiracist Lens. Educ Meas Issues Pract. 2021;40(4):82–90. DOI: 10.1111/emip.12429

[10] Randall J. It Ain’t Near ‘Bout Fair: Re-Envisioning the Bias and Sensitivity Review Process from a Justice-Oriented Antiracist Perspective. Educ Assess. 2023;28(2):68–82. DOI: 10.1080/10627197.2023.2223924

[11] Miller FG. Assessment in action: Toward a more complete and justice-oriented understanding of the social consequences of educational measures. Sch Psychol. 2023;38(3):192–198. DOI: 10.1037/spq0000551 37184961

[12] Brown T, Wyatt J. Design Thinking for Social Innovation. Dev Outreach. 2010;12(1):29–43. DOI: 10.1596/1020-797X_12_1_29

[13] Brown T, Katz B. Change by Design: How Design Thinking Transforms Organizations and Inspires Innovation. 2009; 1st ed. Harper Business.

[14] McLaughlin JE, Wolcott MD, Hubbard D, Umstead K, Rider TR. A qualitative review of the design thinking framework in health professions education. BMC Med Educ. 2019;19(1):98. DOI: 10.1186/s12909-019-1528-8 30947748 PMC6449899

[15] Thomas LR, Nguyen R, Teherani A, Lucey CR, Harleman E. Designing Well-Being: Using Design Thinking to Engage Residents in Developing Well-Being Interventions. Acad Med. 2020;95(7):1038–1042. DOI: 10.1097/ACM.0000000000003243 32101932

[16] Selwyn N. What is ‘Design Justice’? Notes on Costanza-Chock. April 18, 2021. https://data-smart-schools.net/2021/04/18/what-is-design-justice-notes-on-costanza-chock-2020/.

[17] Sloane M, Moss E, Awomolo O, Forlano L. Participation Is not a Design Fix for Machine Learning. In: Equity and Access in Algorithms, Mechanisms, and Optimization. ACM;2022:1–6. DOI: 10.1145/3551624.3555285

[18] Hasan M, Amin S. Design Justice: Why it matters and how you can apply the principles to your work. December 21, 2020. https://archive.researchworld.com/design-justice-why-it-matters-and-how-you-can-apply-the-principles-to-your-work/.

[19] Kakara Anderson HL, Xu X, Edwell A, Lockwood L, Cabral P, Weiss A, Poeppelman R, Kalata K, Shanker AI, Rosenfield J, Borman-Shoap E, Pearce M, Karol C, Schuerer J, Hobday P, O’Connor M, West DC, Balmer DF. How might we build an equitable future? Design Justice, a counternarrative to dominant approaches in medical education. Teach Learn Med. 2024: In Press. DOI: 10.1080/10401334.2024.2404008 39282912

[20] Novak K, Chardin M. Equity by Design: Delivering on the Power and Promise of UDL. Corwin Press, Inc; 2021.

[21] Barab S, Squire K. Design-Based Research: Putting a Stake in the Ground. Journal of the Learning Sciences. 2004;13(1):1–14. DOI: 10.1207/s15327809jls1301_1

