# Supplementary material for: Co-Designing a Justice-Oriented Assessment System in a Pediatric Residency Program: Report from the Designing for Equity in Medical Education Project
Source: Perspect Med Educ. 2025 Apr 7;14(1):141–8. doi: 10.5334/pme.1541 (PMC11987879; doi:10.5334/pme.1541)
Supplement: Supplement 1. — Interview Guide used in a Design Process to Co-Design a more Equitable Prototype Assessment System in Pediatrics. 2024. [file pme-14-1-1541-s1.pdf]

Interview and Observation Guide to Empathize with Users during Step 1 of Design Process to Co-Design a more Equitable Prototype Assessment System in Pediatrics. 2024. Adapted from: Teherani A, Perez S, Muller-Juge V, Lupton K, Hauer KE. A Narrative Study of Equity in Clinical Assessment Through the Antideficit Lens. Acad Med. 2020;95(12S Addressing Harmful Bias and Eliminating Discrimination in Health Professions Learning Environments):S121-S130. doi:10.1097/ACM.0000000000003690

Empathy Map Domain	User Group (s)	Question (s)
Who are our users?	All	Tell me about yourself, your background, identities, and family.
	All	In your own words, how do you feel your perspectives and experiences uniquely contribute to patient care? To the health care team?
	All	Based on your experiences, when you think of the term "assessment", what does that mean to you?
	All	Tell me generally about your experiences with assessment? Either being assessed or assessing others or both.
What do they think?	Learners only	How do your assessments speak to / capture / account for your unique contributions to patient care, if at all?
	Learners only	How do your assessments speak to / capture / account for your growth / learning, if at all?
What do they do?	Learners only	Thinking about your background, identities that you shared at the beginning, how do you feel being your real, authentic self and openly displaying or sharing your identities in this assessment system? [What needs to change in this system to make it easier to openly display and share your identities?]
	Assessors, Leaders	Have you ever thought about the notion of equity when you were assessing learners? If so, have there been times when you felt that you were able to assess leaners in an equitable way? Could you describe an instance?
	Assessors, Leaders	What kind of experience or training have you received in equity in assessment, such as anti-bias training, if any?
	Leaders	What kind of efforts or interventions have you done/implemented in regard to equity? Follow up prompt: how have these efforts been received by faculty? CCC? Residents? Institutional leaders?
	All	This assessment system collects data about your/your learners' performance. What do you think you should be able to do with that data?
	Assessors, Leaders	How does this system help you achieve your teaching or professional goals, if at all?
	All	What about assessment in the residency program doesn't make sense? Is meaningless to you? Confusing?

Empathy Map Domain	User Group (s)	Question (s)
What do they hear?	Leaders, Assessors, Staff	What have you heard from residents about their assessments? What have you heard from attendings and faculty about their assessments? What have you heard from program leadership and your coworkers in the residency program?
	Learners only	Have you ever felt you didn’t receive maybe as many assessments, or as high-quality assessments as others? Have you noticed differences in the number or quality of assessments you receive depending on where you’re rotating, or who’s assessing you?
	All	What have you heard about equity in assessment on Twitter, podcasts, etc. if anything? What do you think about that?
What do they see?	Learners only	Have you ever thought about the notion of equity when you were being assessed during clinical training? If so, were there times during your clinical training in which you felt you were assessed in an equitable way? Could you describe an instance?
	Learners only	Have you have ever received an assessment that you felt was biased, discriminatory, or unjust in this program? If so, what happened? What did you do? Have you had similar or different experiences since? Prompt: what was the process like for following up or reporting that?
	Learners only	Sometimes negative experiences aren’t just about the bad things we experience, but also the good things we don’t experience. Have you ever experienced this? If so, can you tell me about these experiences?
What do they see/hear?	All	Some of the literature in medical education says that equitable assessment systems are primarily founded on "real time, frequent, transparent, and specific feedback" and "Positive relationships with clinical supervisors." What would you add to, subtract, or maybe modify about that description? Given that description of an equitable assessment system that you just provided, how is this assessment system doing? How does equity show up here in this assessment system, if at all?
Closing question	All	Is there anything else related to your experiences of equity that you would like to share?
Closing question	All	Are there any topics or questions that we didn't get to today that would be important for us to know as we do this project?
