# Supplementary material for: Co-Designing a Justice-Oriented Assessment System in a Pediatric Residency Program: Report from the Designing for Equity in Medical Education Project
Source: Perspect Med Educ. 2025 Apr 7;14(1):141–8. doi: 10.5334/pme.1541 (PMC11987879; doi:10.5334/pme.1541)
Supplement: Supplement 2. — Users and Outputs for Each Step of a Design Process to Co-Design a more Equitable Prototype Assessment System in Pediatrics. 2024. [file pme-14-1-1541-s2.pdf]

Supplement 2: User Information and Outputs for Each Step of a Design Process to Co-Design a more Equitable Prototype Assessment System in Pediatrics. 2024.

User Participants by Step in Design Process:

		Users			
		Resident learners	*Faculty assessors; Faculty mentors*	*Program staff*	*Educational leaders*
	Role	Primary recipients of assessments	Deliver and interpret assessments	Manage administrative workflows of assessment	Program Director, Associate Program Directors, Directors of Curricula, Chief Residents
Steps of the Design Process	*Step 1 (Empathize)*	Interviewed (4)	Interviewed (5); observed (2)	Interviewed (2); observed (1)	Interviewed (4)
	Step 2 (Define)	Session participants (11)	Session participants (13)	Session participants (3)	Session participants (6)
	Step 3 (Ideate)	Session participants (12)	*0*	*0*	Session participants (1)
	Step 4 (Co-create)	Session participants (16)	*0*	*0*	Session participants (1)
	Total participants:	43	20	5	12

Demographics of Participants who Completed Optional Demographic Survey (n=36)

**Demographic**	**Percent (n)**
Race and ethnicity[1]	
Asian	16.7% (6)
Black/African American	8.3% (3)
Hispanic/Latine	11.1% (4)
Underrepresented in Medicine[2]	19.4% (7)
White	69.4% (25)
One or more races/ethnicities	11.1% (4)
Gender	
Female	77.7% (28)
Male	19.4% (7)
Nonbinary and gender non-conforming	5.6% (2)
Disability	
Disabled	16.7% (6)
Non-disabled	83.3%
First-generation	
Attend college	19.4% (7)
Attend medical school	69.4% (25)

Outputs:

Step 1 output: empathy maps. Shown: example empathy map for resident learners.

Resident Leaner

EMPATHY Map

Designed for Site : CHOP

Designed by : HA

Date: 9/7/23

Version : 2

Who are we empathizing with?

"People have treated me with closed minds in health care spaces and so I tend to be like.. I'm just gonna listen to what you're telling me and try to understand where you're coming from. Just because I know how it feels to not have people listen to you or to assume that you feel certain way."

"I have become more confident - honestly, from feedback and assessment of what people tell me my strengths are. And that has made me more confident to kind of lean into those things, lean into my strengths and be more myself in that way. And not pretend to be someone else because I've learned that just doesn't play to your own strengths and you can't be anybody else."

What do they hear?

"This is the biggest thing, you get feedback in a written assessment that, if it's written, that means it's gone to the program leadership. And it's something that that person never discussed with you in person… The assessment system can be very one way and final. You can't reach back. You could. But that's a lot of activation energy to reach back out. Especially if it's something that 1) you don't agree with or 2), you might view it as negative. That's a lot to ask a trainee. That's what I hear from the resident."

"It feels good when someone's like, "you connect with the family." I was stoked about that [assessment]… I was also thinking, was it just because of my trans identity, though? I doubted myself, like, did I really earn this?"

"[assessment data] was never really brought up to me until my exit interview where the assistant program director was like, You started off with these numbers and now you're these numbers and so you have improved and I was like, great, I would hope so."

What do they say?

"I think sometimes attendings can kind of project their shortcomings unto us a little bit. Like I've received evaluations in the past that said my differential wasn't broad enough and I prolonged the stay of a patient or I you know, discharged a patient before they were ready and like ultimately like neither of those things are our responsibility, the buck stops with the attending and so I feel that was a little bit of unjust to say that those were my faults or my shortcomings when really like that's a shortcoming of whatever attending was on service."

What do they need to do?

"[One of] the attendings had said, the way that you said the sentence could have been better. I appreciated that mostly because that had happened multiple days before and he remembered it to tell me and so I was like, oh, he's paying attention to my communication style and skills. When I was an intern on the complex care service, I was with an attending who knew me pretty well. And I think her feedback and evaluation was very tailored to like what was important to me. It kind of helps me build confidence in areas that I was working on, like no, no, like you talk to families on rounds in a nice way and I want you to keep working on doing that. So I think just assessing what's important and like encouraging skill building in that area."

What do they see?

"There is a question in our evaluations about "Respects others of different identities" Etc…. I don't think that's a good question to assess like is my identity helping to advance care and things like that. I mean quite frankly. I think the questions that we have on our evaluations are not ideal…the questions that they ask are really pretty standard and I don't think that they get at a lot. They're very surface level and they don't necessarily get at like who we are as doctors."

"My pronouns are like a soup in my written evaluations that I have. I'm like, who are we talking about? Are you talking about me? It's like they're not even referring to me. I think sometimes that's been confusing and just like, wow, I've really worked with this attending for 2 weeks and they didn't notice my pronoun?... When I assess medical students, their pronouns are on the top of the form."

"Where it gets really hard is when you don't see yourself in your evaluations, and especially when you get evaluations that are like very contrary to your beliefs like your values that you hold that you hold close to yourself. I think that can be very distressing and I think it is very distressing for a lot of people. Oftentimes these are like attendings you only work with for like a day like a couple of hours and then like maybe I was having a bad day. Maybe they were having a bad day. Maybe like maybe everyone's having a bad day. Maybe it was really busy. I think that can be very distressing and they can kind of make you question who you are as a person when you receive this feedback that doesn't align with who you see how you see yourself in your head."

What do they do?

"Rounds feels very performative.. It feels like you can't be as much yourself… a lot of times the senior resident has already looked at all the numbers, the attending has also already reviewed all the numbers. So then whoever is presenting is just saying the numbers. Just for the performance because that is sometimes the only time of the day that you see your attending in person… I think if we got back to less of performance on rounds and just using your interpersonal skills and sharing with families. I think everyone has really great unique styles of how they communicate with patients and families and that's where they could probably shine the most and learn from the assessment of their personal level of growth."

Step 2 output: Lists of pains and gain. Shown: program leadership and staff pains

Program Leadership / Staff PAINS:

"We talk about people, but we don't have enough data:" it's hard to make decisions and support learners with very limited information: ***"Unless one of us sees them, we don't know… You would never be able to tell from the evaluations."***

"I worry that our view is too narrow:" leadership and staff worry that they're not seeing everyone who is struggling; worry about learners who are not visibly minority or marginalized but are experiencing inequity

"I don't think they know what the Milestones or EPA's mean:" the numbered scales are hard to interpret, leaders worry about the quality of data

"The programs do not work together:" important systems that must be used with New Innovations (Qgenda, GME databases, etc.) do not connect with it: ***"it all has to be done manually, which leads to errors"***

"I don't challenge it:" when some staff and leaders notice inequitable assessments or errors, ***"I don't always feel safe to say anything"***

Step 3 output: Design principles

We are pediatric residents, faculty, staff, and leadership at SITE.
We are working towards an equitable assessment system, which means:

We see assessment and feedback as interdependent, mutual, and shared endeavors; therefore, we hold each other accountable and support each other, aware of impacts and consequences on each other

We ensure there are clear, transparent processes so that each person knows what they need to do and how to do it

We practice trustworthy, respectful behaviors so that each person is seen for who they are and who they can be – and sees each other for who they are and can be

We nurture many diverse ways of being and becoming a pediatrician so that everyone has freedom to flourish
